# MGIS: managing banana (*Musa* spp.) genetic resources information and high-throughput genotyping data

**DOI:** 10.1093/database/bax046

**Published:** 2017-06-11

**Authors:** Max Ruas, V. Guignon, G. Sempere, J. Sardos, Y. Hueber, H. Duvergey, A. Andrieu, R. Chase, C. Jenny, T. Hazekamp, B. Irish, K. Jelali, J. Adeka, T. Ayala-Silva, C.P. Chao, J. Daniells, B. Dowiya, B. Effa effa, L. Gueco, L. Herradura, L. Ibobondji, E. Kempenaers, J. Kilangi, S. Muhangi, P. Ngo Xuan, J. Paofa, C. Pavis, D. Thiemele, C. Tossou, J. Sandoval, A. Sutanto, G. Vangu Paka, G. Yi, I. Van den houwe, N. Roux, M. Rouard

**Affiliations:** 1 Bioversity International, Parc Scientifique Agropolis II, 34397 Montpellier Cedex 5, France; 2 South Green Bioinformatics Platform, Montpellier, France; 3 CIRAD, UMR AGAP 34398 Montpellier Cedex 5, France; 4 USDA-ARS-Tropical Agriculture Research Station, Mayaguez, Puerto Rico; 5 University of Kisangani, Kisangani (UNIKIS), Democratic Republic of Congo; 6 Taiwan Banana Research Institute (TBRI), Chiuju, Pingtung, Taiwan, Republic of China; 7 Department of Agriculture, Fisheries and Forestry, Queensland Government (DAFF South Johnstone), Brisbane, Australia; 8 Institut National pour l'Etude et la Recherche Agronomiques (INERA), Democratic Republic of Congo; 9 Centre National de la Recherche Scientifique et Technologique (CENAREST), Libreville, Gabon; 10 Institute of Plant Breeding (IPB), University of the Philippines (UPLB), Los Baños, Philippines; 11 Bureau of Plant Industry (BPI) - Davao National Crop Research and Development Center, Davao City, Philippines; 12 Centre Africain de Recherche sur Bananes et Plantains (CARBAP), Njombe, Cameroon; 13 Bioversity International, International Musa Germplasm Transit Center (ITC), KULeuven, Leuven, Belgium; 14 Agricultural Research Institute (ARI) Maruku, Bukoba, Tanzania; 15 National Agricultural Research Organization (NARO), Mbarara, Uganda; 16 Fruit and Vegetable Research Institute (FAVRI), Hanoi, Vietnam; 17 National Agricultural Research Institute (NARI), Laloki Papua, New Guinea; 18 CRB Plantes Tropicales, CIRAD INRA – Neufchâteau, Guadeloupe, France; 19 Centre National de Recherches Agronomiques (CNRA), Abidjan, Cote d’Ivoire; 20 Institut National de Recherche Agronomique du Bénin (INRAB), Cotonou, Bénin; 21 Corporación Bananera Nacional S.A (CORBANA), San José, Costa Rica; 22 Indonesian Centre for Horticultural Research and Development (ICHORD), Bogor, Indonesia; 23 Institute of Fruit Tree Research (IFTR), Guangdong Academy of Agricultural Sciences (GDAAS), Guangdong, China

## Abstract

Unraveling the genetic diversity held in genebanks on a large scale is underway, due to advances in Next-generation sequence (NGS) based technologies that produce high-density genetic markers for a large number of samples at low cost. Genebank users should be in a position to identify and select germplasm from the global genepool based on a combination of passport, genotypic and phenotypic data. To facilitate this, a new generation of information systems is being designed to efficiently handle data and link it with other external resources such as genome or breeding databases. The *Musa* Germplasm Information System (MGIS), the database for global *ex situ*-held banana genetic resources, has been developed to address those needs in a user-friendly way. In developing MGIS, we selected a generic database schema (Chado), the robust content management system Drupal for the user interface, and Tripal, a set of Drupal modules which links the Chado schema to Drupal. MGIS allows germplasm collection examination, accession browsing, advanced search functions, and germplasm orders. Additionally, we developed unique graphical interfaces to compare accessions and to explore them based on their taxonomic information. Accession-based data has been enriched with publications, genotyping studies and associated genotyping datasets reporting on germplasm use. Finally, an interoperability layer has been implemented to facilitate the link with complementary databases like the Banana Genome Hub and the MusaBase breeding database.

**Database URL:** https://www.crop-diversity.org/mgis/

## Introduction

The collection, conservation, characterization and breeding of cultivated plants and their wild relatives contribute to the preservation of biological diversity and are essential components in ensuring food security. Information systems for plant germplasm collections or genebanks (e.g. Genesys www.genesys-pgr.org, GRIN-Global www.ars-grin.gov/npgs) provide documentation on a large number of Plant Genetic Resources (PGR). In addition, these information systems allow users to request PGRs, including seeds, *invitro* plantlets, leaves and other types of samples. Conservation and further use of PGRs rely on information systems describing the germplasm material held in collections. For many crops, insufficient genetic information is available on the holdings in genebanks. Furthermore, these systems contain little genomic data, in spite of their usefulness in characterizing genetic resources. Conversely, crop genomic databases focus on a small number of genotypes restricted to a reference genome sequence for a particular species or crop (1–3) or a collection of reference genomes to facilitate comparative genomic studies (4–6). Information on the selected sequenced genotypes is often considered secondary data, and links to the original germplasm are frequently missing or included only in the publication, which can hamper researchers wishing to perform subsequent analyses on the same germplasm. These two types of resources have traditionally been managed with distinct tools and by different communities, although some recent developments are now proposing to create interoperability between those different datasets (7, 8). Next-generation sequencing (NGS) has the potential to change the way scientists deal with genetic resources by unlocking genetic diversity stored in genebanks (9, 10). This technology also allows genebank users to select germplasm material based not only on passport and phenotypic data, but also on genomic information. However, NGS raises several unique challenges, including managing the volume and diversity of generated sequences, providing appropriate storage facilities and developing analytical and graphical visualization tools (11–13).

Bananas (*Musa* spp.) are an important staple crop for global food security. They are susceptible to many pests and diseases (e.g. Panama disease/Fusarium wilt) which greatly limits global production (14). Although commercial banana production is dominated by only a few genotypes, around the world collections of *Musa* germplasm contain >15 000 accessions (i.e. unique samples in a germplasm collection) distributed among 56 collections (15). These collections represent a wide range of phenotypic variation and genome constitutions. Unraveling and exploiting genetic diversity in banana germplasm collections is critically important as long-term sustainable crop cultivation depends on this diversity in order to adapt to the ever-changing agro-environment. Here, we report on the latest developments of the *Musa* Germplasm Information System (MGIS). MGIS is the largest database for banana genetic resources and includes passport and characterization data, voucher images and genomic-based marker information generated for a large number of accessions. Data associated with accessions in MGIS will help identify important agricultural traits of banana and thus support targeted analyses and distribution of diverse germplasm to researchers, breeders and farmers.

## Database content

### Germplasm data

**Figure 1.**
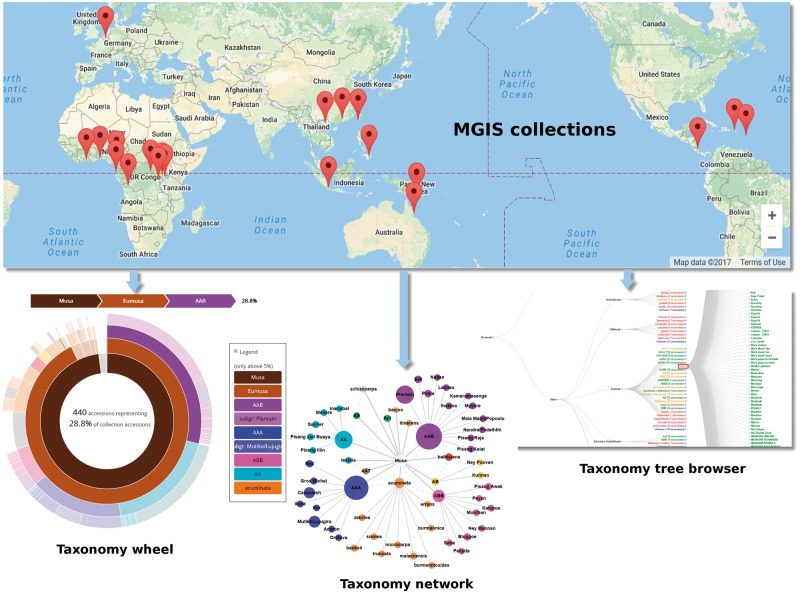
Overview of the germplasm collections in MGIS and associated browsers to explore banana diversity via the taxonomy wheel, the taxonomy tree and the taxonomy network. All of them provide an easy way to navigate within the accessions.

The MGIS crop-specific database was originally developed with the objective to collect and share publicly all available information on the accessions held by *ex situ Musa* collections worldwide (Figure 1). It contains key information including passport data, botanical classification, morphotaxonomic and phenotypic descriptors, ploidy and digital voucher images. It is compliant with the Multi-Crop Passport Descriptors (MCPD) (16) which have been widely used as the international standard to facilitate germplasm passport information exchange. Banana germplasm are identified by accession numbers (i.e. unique identifier in a collection) that will be complemented soon by Digital Object Identifiers (DOI) following the specifications of the International Treaty on Plant Genetic Resources for Food and Agriculture (ITPGRFA) (http://www.fao.org/plant-treaty/areas-of-work/global-information-system/faq/en/).

Currently, MGIS maintains information related to 4587 accessions from 21 collections (Table 1). The main source of information and most diverse set of *Musa* germplasm available for distribution internationally is managed by Bioversity International’s International *Musa* Germplasm Transit Center (ITC) and hosted at the Katholieke Universiteit Leuven (KU Leuven) in Belgium. ITC accessions that are certified disease-free can be requested directly via the MGIS website. Users can also download the Standard Material Transfer Agreement (SMTA) that is generated automatically online and required for exchange of plant material regulated under ITPGRFA (www.fao.org/plant-treaty/en/).

**Table 1.** List of banana collections and status of data sharing in MGIS (as of March 2017)

*Collections in MGIS (DSA signed and data updated)*			
*Country*	Acronym	Institute	No. of accessions
*Australia*	DAFF South Johnstone	Department of Agriculture, Fisheries and Forestry, Queensland Government	42
*Belgium*	ITC	Bioversity International Musa Germplasm Transit Centre	1527
*Benin*	INRAB	Institut National de Recherche Agricole	19
*Cameroon*	CARBAP	Centre Africain de Recherche sur Bananes et Plantains Station de Recherches Agronomique	356
*China*	IFTR/GDAAS	Institute of Fruit Tree Research, Guangdong Academy of Agricultural Sciences	217
*Republic of China*	TBRI	*Taiwan* Banana Research Institute	225
*Congo, DRC*	INERA (Mvuazi)	Institut National pour l'Etude et la Recherche Agronomiques	57
*Congo, DRC*	INERA (Mulungu)	Institut National pour l'Etude et la Recherche Agronomiques	36
*Congo, DRC*	UNIKIS-FS	Faculty of Sciences, University of Kisangani	109
*Costa Rica*	CORBANA	Corporación Bananera Nacional S.A.	108
*Gabon*	CENAREST	Centre National de la Recherche Scientifique	36
*Guadeloupe(France)*	CIRAD-INRA	CRB Plantes Tropicales, INRA CIRAD	381
*Indonesia*	ICHORD	Indonesian Centre for Horticultural Research and Development	306
*Ivory coast*	CNRA	Centre National de la Recherche Agronomique	71
*Papua New Guinea*	NARI-LALOKI	Southern Regional Centre – Laloki	146
*Puerto Rico*	USDA-TARS	United State Depart. Of Agriculture, Tropical Agriculture Research Station	152
*Philippines*	BPI-DNCRDC	Bureau of Plant Industry - Davao National crop research and development center	86
*Philippines*	UPLB	University of the Philippines Los Baños	69
*Tanzania*	ARI-Maruku	Agricultural Research Institute Maruku	118
*Uganda*	NARO	National Agricultural Research Institute, PGR Unit	442
*Vietnam*	FAVRI	Fruit and Vegetable Research Institute	84
*Collections not yet in MGIS (but DSA signed)*			
Country	Acronym	Institute	
*Central African Republic, CAR*	ICRA	Institut Centrafricain de Recherche Agronomique	
*Colombia*	FEDEPLATANO	Federacion nacional de Plataneros de Colombia	
*Comores*	INRAPE	Institut National de la Recherche pour l'Agriculture, la Pêche et l'Environnement	
*Cuba*	INIVIT	Instituto de Investigaciones de Viandas Tropicales	
*Ghana*	CSIR-CRI	Council for Scientific and Industrial Research - Crops Research Institute	
*Malaysia*	MARDI	Malaysian Agricultural Research and Development Institute	
*Madagascar*	CENRADERU	Centre National de la Recherche Appliquée au Développement Rural	
*Nigeria*	NIHORT	National Horticultural Research Institute	

The level of passport data completeness is assessed by the Passport Data Completeness Index (PDCI) (17), adapted to the specificities of *Musa* datasets in MGIS (Supplementary Figure S1) and thus noted as PDCI_m_ (m for *Musa*). This index is built on the parameters required to calculate the PDCI as implemented in Genesys, but considers eight additional fields such as type of material and previous locations (Supplementary Table S1). The index helps partner genebanks to identify deficiencies in their passport data and to improve associated information. The PDCI_m_ is publicly available on MGIS for the passport data of accessions conserved in the ITC collection, while the indices of data from other collections are only visible to their respective curators as an internal tool for data quality management.

The main target users of the MGIS database are: (i) banana germplasm curators requiring a global system for sharing, comparing and managing of data in their own collections, (ii) researchers, breeders and direct users of the germplasm who select the most documented material for various types of experiments and/or production and (iii) general users looking for reference information associated with characteristics of cultivars, crop wild relatives or improved varieties at the accession level.

**Figure 2.**
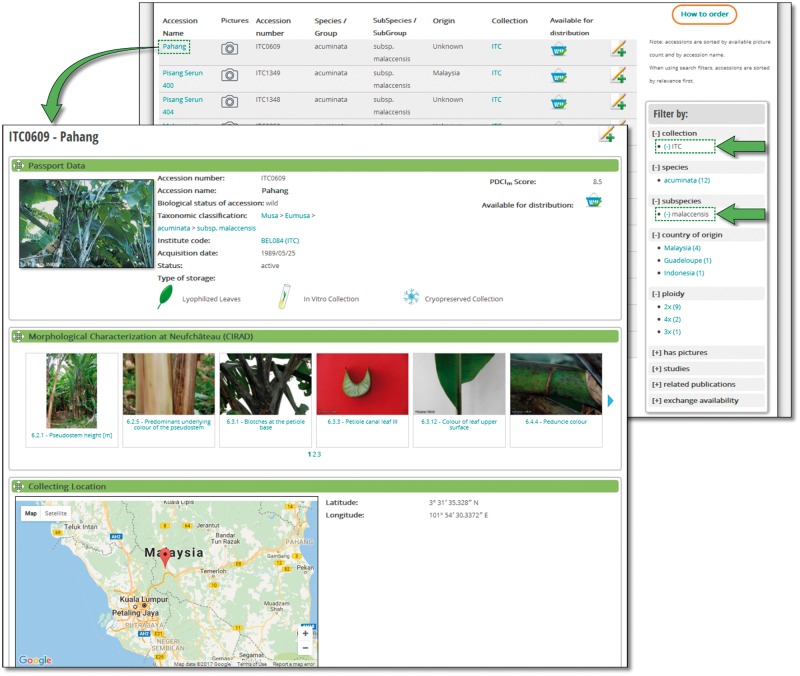
Accession search and accession page. A list of accessions can be filtered by a wide range of parameters automatically updated with a number of elements. A typical accession page displays various sections such as passport data, previous location and collecting sources, genetic integrity, morphological and molecular characterization and publications.

**Figure 3.**
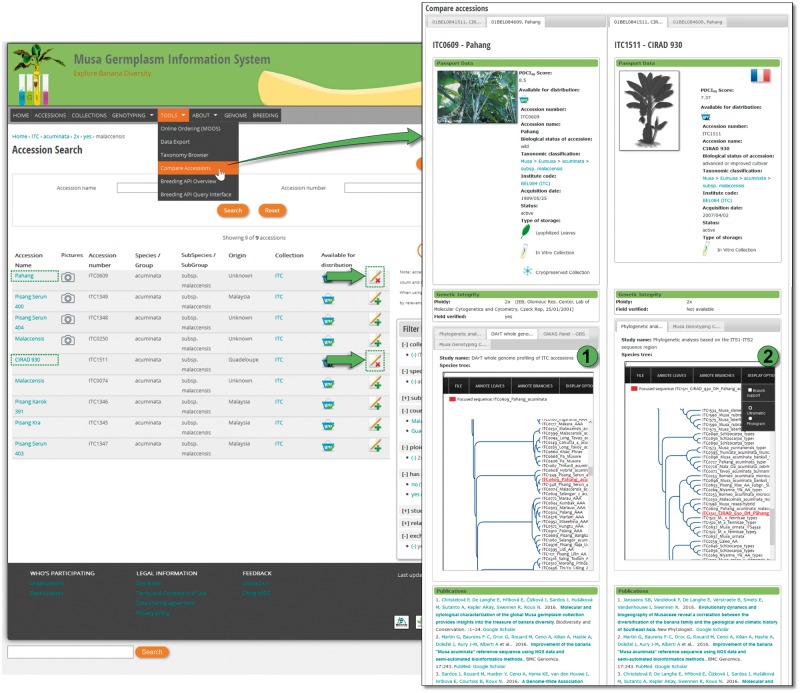
Comparison of ‘Pahang’ and ‘DH Pahang’ (cirad 930) accessions. Once selected, accessions can be compared in order to check morphological differences and genetic tree topologies. Here, ‘Pahang’ parent of ‘DH Pahang’ is logically found to be the most similar accession in two different genotyping studies.

MGIS helps users build customized queries, facilitates data export, locates alternative sources of banana germplasm and identifies the most appropriate accessions for users’ needs (Figure 2). Users can access information regarding the country of origin of the germplasm, its availability, ploidy level, genomic constitution and genetic integrity of accessions verified in the field (18) as well as a set of standardized morphological descriptors with associated voucher images. A recently added function allows users to compare and contrast two accessions side-by-side, highlighting the differences in phenotypic characters and/or, when available, genetic relatedness based on diversity trees (Figure 3).

### Scientific literature

Monitoring the use of PGR is one of the many responsibilities of germplasm collection curators, but often this information is not reported or relayed back to the genebanks. Information on the past use of accessions is important for traceability purposes. It can also be particularly insightful for subsequent experiments or even to prevent duplicate studies. In order to track accessions used in research experiments, we developed a way to link accessions to research publications that involved *Musa* PGR. We initially focused on the use of germplasm in the ITC and performed a detailed search of relevant scientific literature using several bibliographic search engines (e.g. MusaLit www.musalit.org/, Google Scholar scholar.google.fr/) by using a set of keywords that indicated that the material was obtained from the ITC. As of December 2016, 1085 ITC accessions recorded in MGIS (based on 110 publications) have been quoted in at least one peer-reviewed publication (Supplementary Figure S2). Additional publications associated with other collection accessions in MGIS, such as research published on accessions from the USDA National Plant Germplasm System and CIRAD collections, were linked in MGIS as well. However, it would require further work and detailed interactions with the curators of those germplasm collections to complete an exhaustive list of research publications.

It is interesting to investigate the reasons for variations in the published use frequencies of a particular accession. We found some wild accessions, such as ‘Calcutta 4’ (ITC0249) which is the most requested and studied accession in the literature. ‘Calcutta 4’ has contributed to many genomic resources (BAC libraries, ESTs, etc.) and has been extensively used in breeding programs. Another prominent accession from MGIS in the literature is ‘Pahang’ (ITC0609), which happens to be the parent of ‘DH Pahang’, the double haploid accession used to generate the *M. acuminata* reference genome sequence (19). Not all germplasm in the MGIS has been associated with requests, evaluation and/or published literature and several reasons for this might exist. One of the major limitations in the use of ITC germplasm is that 32% of the accessions are not available for distribution due to the presence of the integrated form of banana streak virus (BSV) in the DNA of accessions containing the B genome. The lack of availability of these BSV infected accessions may evolve in the near future due to recent changes in ITC distribution policies (20). For the remaining accessions with no associated published scientific literature, insufficient documentation is suspected, as many of these accessions do not have a comprehensive set of images and/or characterization data. However, this information has to be examined in a broader context because germplasm use is likely more extensive than what is reported in scientific literature.

### Genetic diversity studies

**Figure 4.**
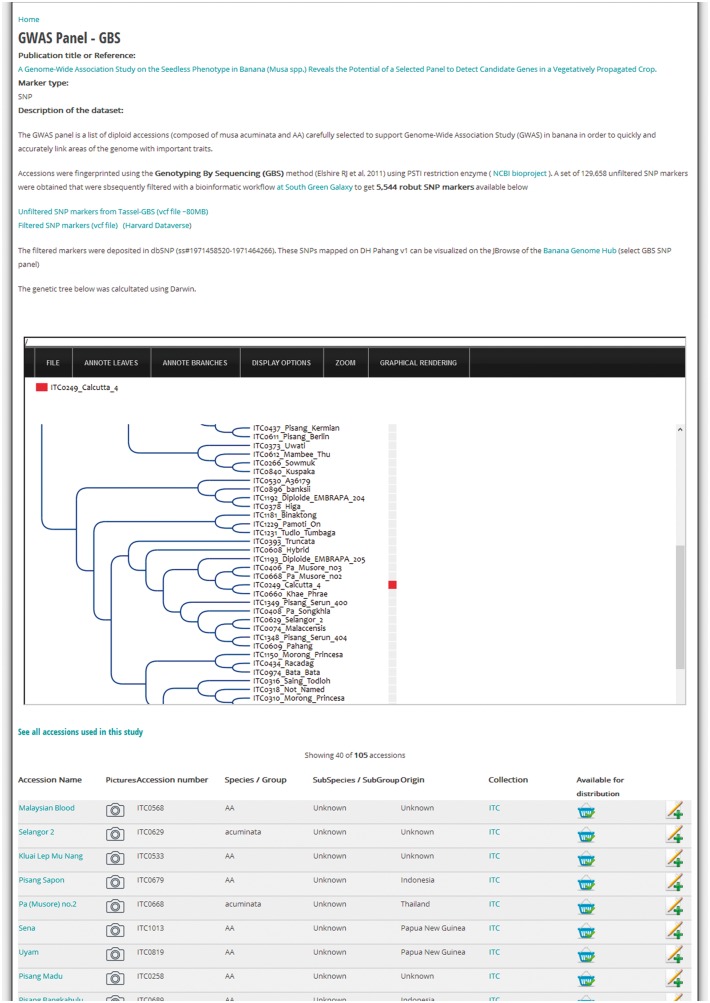
Example of a diversity study page. Screenshot of a MGIS genotyping data content page (i.e. GWAS study) including a gene tree powered by the InTreeGreat viewer. Lists of accessions with passport data are accessible and material can be ordered online. Additional metadata are indicated such as the publication, marker type and dataset marker availability in public databases (e.g. NCBI SRA or dbSNP).

Research and subsequent publication might involve genotyping a set of *Musa* spp. accessions received from ITC. In that case, it is relevant to highlight the results of the diversity analyses to complement the morphological data or fill gaps in the passport data. Therefore, MGIS includes a set of studies based on genotyping with microsatellite markers (21), DArT markers (22) and single-nucleotide polymorphisms (SNP) from Genotyping-by-Sequencing (GBS) for several hundred accessions (23). This is a convenient way for users to identify the accession of their choice in a dendrogram or diversity/phylogenetic tree and eventually confirm its taxonomic classification before requesting germplasm. For instance, Figure 4 illustrates how to access such a tree obtained from a Genome-Wide Association Study (GWAS) on the seedless trait in *Musa* (24). Below the tree, a table lists the 105 accessions comprising the panel used for the study, which can be requested through MGIS to perform further GWAS on other traits. In the website administration back-end, we developed an efficient method to generate a list of accession numbers with publications and diversity trees in Newick format (evolution.genetics.washington.edu/phylip/newicktree.html) that will automatically tag any accession with the associated study and redirect them to the detailed study page. As a result, users can compare the genetic profiles of accessions in the same or different studies inserted in the database.

### Genomics-based data

Since the sequencing of the *M. acuminata* subsp. *malaccensis* double haploid ‘DH Pahang’ genome (19), a high-quality reference genome sequence with annotated genes is available (25, 26). NGS technologies have boosted research that helps better understand banana genetic diversity, generating millions of SNP markers computed using bioinformatics methods. Currently, more than one-third of the 1527 accessions of the ITC collection have been analysed by high-throughput genotyping such as GBS or RADSeq methods (23) and some studies have been initiated with those datasets to perform trait-gene associations such as GWAS (24).

**Figure 5.**
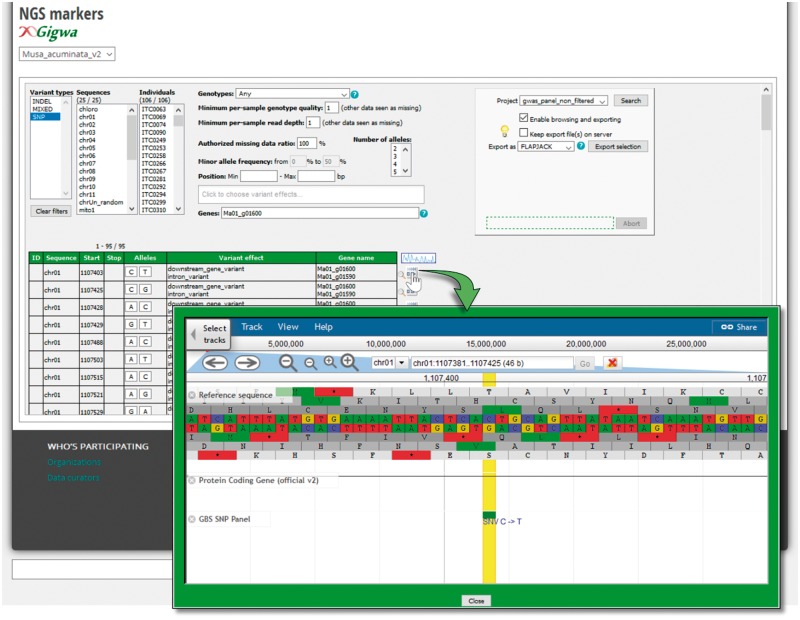
Genotyping search page powered by the GIGWA system. SNPs and InDels can be filtered by a wide range of criteria (chromosomes; Minor Allele Frequency (MAF), missing data, genes, gene effect, etc.) and exported in various formats. Markers can be also located on their gene as provided by the Banana Genome Hub.

In order to manage and make available these large datasets to users, we implemented a genotyping module in close interaction with the GIGWA-Genotype Investigator for Genome-Wide Analyses (27) project that aims to provide efficient and scalable tools to handle high-throughput genotyping data. MGIS has been extended with interfaces to query SNP datasets by setting up a dedicated instance of GIGWA that has been integrated with MGIS (Figure 5). With this function, users can filter a large quantity of SNP markers and export these in the format of their choice including VCF, GFF, DARwin, IGV (28) and Flapjack (29). The system is compatible with multiple reference genomes or assembly versions, as is the case in banana, with the recent release of an improved assembly of the reference genome (25).

### System architecture

**Figure 6.**
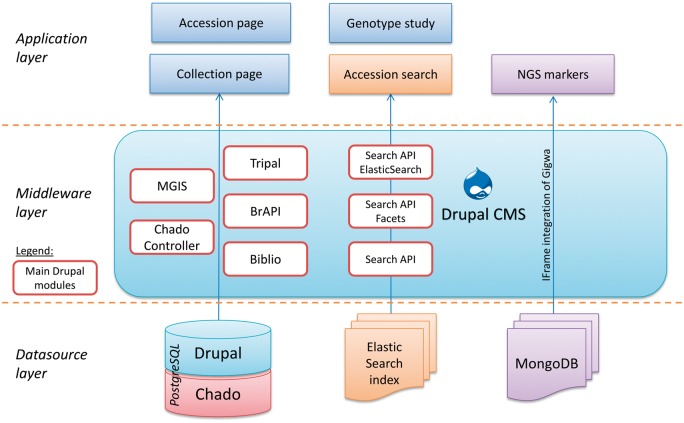
Software architecture of MGIS.

MGIS is implemented with the Drupal Content Management System (www.drupal.org/) using the Tripal module (30, 31) to work with the standard Chado database schema (32) (Figure 6). This solution based on GMOD tools has been adopted and applied to plant genome resources such as Rosacea (33), Cotton (34), *Medicago* (35), *Arabidopsis* (1), Banana (i.e. genome hub) (26) and Coffee (36). This open source community has also recently been extended and documented as a case study to manage plant germplasm and plant breeding data (8, 37). One reason for Drupal's success is that it is extensible, and that new modules can be developed to meet specific needs. Both shareable and in-house Drupal modules have been developed for MGIS. In order to manage Chado data access levels, modification history and data integrity checks, we developed an open source module called the Chado Controller (38). This module relies on another extension that we developed, the Tripal Multi-Chado module, (www.drupal.org/project/tripal_mc) which also enables the generation of Chado sandboxes for collection updates. The sandboxes are clones of the live database that can be safely altered by germplasm collection curators in order to validate their updates and later propagate them on the live database. An additional in-house extension called MGIS has been developed in order to solve some MGIS-specific needs such as germplasm requests, comparison, verifications and data visualization (Figure 1).

To manage faceted searches (i.e. a search query with multiple factors, for example, country of origin, ploidy and exchange availability), we used ElasticSearch (/www.elastic.co/) associated to the Drupal Search API ElasticSearch module (www.drupal.org/project/search_api_elasticsearch) coupled with Search Facets module (www.drupal.org/project/faceted_search), which provided filtered accession searches on various criteria such as the collection, country of origin, taxonomy or genotyping study (Figure 2). We enabled a feature of ElasticSearch called fuzzy search that allows an approximate text matching in accession name queries. This function is particularly useful in deciphering the many variations of vernacular names in banana. With these functions, MGIS has become a very user-friendly and efficient germplasm search interface.

The publication management feature has been implemented using the Biblio module (www.drupal.org/project/biblio) that we extended to enable mapping with a list of accessions. It provides a comprehensive publication reference with links to article details, authors or Google scholars. Diversity trees are visualized using InTreeGreat developed by the South Green platform (39), which provides an advanced graphical interface to browse trees, and is embedded using an iframe to preserve the style of the MGIS website. To address the big data volume (i.e. NGS), we adopted the GIGWA system (27) to manage high-throughput genotyping data. The application has been installed on the dedicated server and is included in the Drupal front-end via an iframe (Figures 5 and 6).

### Database interoperability

As SNPs are mapped on the reference genome in close proximity to genes of interest, we strengthened the links with the Banana Genome Hub (banana-genome-hub.southgreen.fr) (26) by adopting same style interfaces and making crosslinks. Datasets were also provided to SNiPlay (sniplay.southgreen.fr) (40) to foster additional analyses. In addition, linking genotyping data with breeding data stored in MusaBase (musabase.org) has been a valuable objective to be able to cross data between breeding trials, pedigree and wild material or landraces distributed by germplasm collections. To facilitate the interoperability, we developed web services compliant with the plant Breeding API, BrAPI (docs.brapi.apiary.io) that are detailed in MGIS at the following address (www.crop-diversity.org/mgis/brapi/overview). The current implementation as an open source Drupal module (www.drupal.org/project/brapi) enables us to link genebank material in MGIS that are used as parents of crosses and to list their progenies in MusaBase. Finally, once the data has been collected, structured and curated, quality data on passport data for the ITC collection are transferred to Genesys (www.genesys-pgr.org/wiews/BEL084), the global portal to information on Plant Genetic Resources for Food and Agriculture (PGRFA). 

### Conclusions and perspectives

Partnerships and collaborations are at the heart of the MGIS *modus operandi* for collecting and exchanging data. By sharing data, germplasm collections help to define the global status of the *ex situ* banana diversity conserved worldwide and thus support rationalization and gap analyses. The number of participating germplasm collections is expected to grow steadily with the signing of more Data Sharing Agreements (DSA) in which both parties (Bioversity International and partners’ collections) engage to deliver the most accurate *Musa* germplasm information available. Contributing to MGIS with data benefit from a wide range of advanced tools applied to their collection.

Efforts are continuously being made to improve MGIS data content. An interdisciplinary team composed of germplasm curators, geneticists, bioinformaticians and computer scientists working with taxonomists (e.g. MusaNet‘s taxonomy advisory group) have collaborated in this project to (i) enhance the quality of the data at the collection level leading to the identification of potential inconsistencies in passport data in global banana collections, (ii) implement an adapted graphical interface for the community in MGIS, and (iii) increase the connectivity of MGIS with complementary datasets (e.g. genomics).

Regarding the latter, GBS analyses are also an integral part of the research and management of genetic resources. Genomic-based analyses play an increasingly important role in understanding and exploiting the genetic variation of crop diversity maintained in genebanks. And as such can contribute to enhance the productivity, sustainability and resilience of banana cultivars and their agricultural systems. Finally, although there is an increasing amount of phenotypic information available, one of the key limiting factors in its use has been the lack of standard nomenclature used to describe crop development and agronomic traits. In order to facilitate access to harmonized data held in a range of databases, we will adopt the Crop Ontology scheme/methods (41) to manage interoperability between phenotyping experiments on banana germplasm and design interfaces for the management of phenotyping data.

## Supplementary data

Supplementary data are available at *Database* Online.

## Supplementary Material

Supplementary Data
