# Supplementary material for: MGIS: managing banana (Musa spp.) genetic resources information and high-throughput genotyping data
Source: Database (Oxford). 2017 Jun 11;2017:bax046. doi: 10.1093/database/bax046 (PMC5502358; doi:10.1093/database/bax046)
Supplement: Supplementary Data [file bax046_Supp.docx]

**Supplementary Figure 1 : Distribution of the ITC accessions by PDCIm score as of March 2017.** The Passport Data Completeness Index for Musa reflects the quality of documentation for a banana plant. Most of them are between 6 and 7.

**
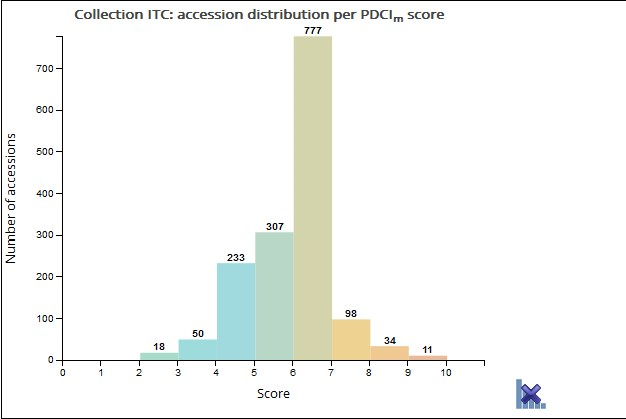
**

**Supplementary Figure 2 : Reported use of ITC accessions in peer-review publications as of March 2017.** This diagram illustrates the reported use of the banana genetric resources. 437 accessions were reported once while more than 10 accessions were extensively used in research studies (>28 publications).

**
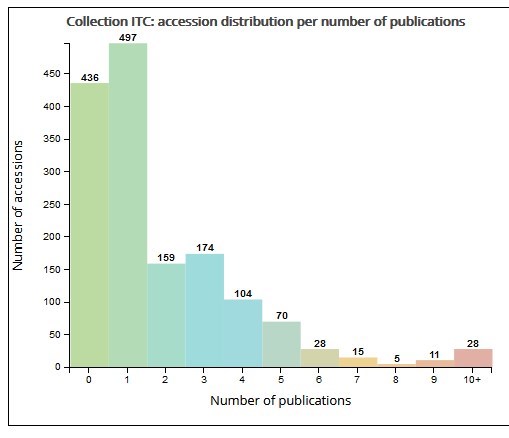
**

**Supplementary Table 1: method of calculation of the PDCIm (aka Relative Data Item Count).** This index was based on the Passport Data Completion Index (PDCI) (van Dooijeweert and Menting , 2008, van Hintum *et al*,2011) but slightly modified in order to reduce the complexity of the weighting used in PDCI and thus make easier to add new fields. Mapping of the field between PDCI and PDCIm are indicated in the “PDCI equivalent” column and new fields are labeled n/a. Field names are based on the Multi Crop Passport Descriptor standards. PDCIm=(actual score/maximum score) x 10.

| **PDCIm** | **Description** | **PDCI equivalent** | **Scoring value** | **Population type^[[1]](#footnote-1)^** | **Comment** |
| --- | --- | --- | --- | --- | --- |
| TYPITO | Type of material received 1 = In Vitro Plant, 2 = sucker, 3 = Seed, 4 = Bud, 5= Other | n/a | 1 | All |  |
| SAMPSTAT | Population type: 1= Wild, 2= Trad. cultivar, 3= Breeders material, 4= Modern cultivars, 5=Other. | SAMPSTAT | 1 | All |  |
| TYPPRL | Type of Previous location 1 = Collection, 2 = Collecting mission 3= Unknown | n/a | 1 | All |  |
| ACQDATE | Introduction of accession date | ACQDATE | 1 | All |  |
| ORIGCTY | Country code of origin | ORIGCTY | 1 | All |  |
| GENUS | Taxon code (was converted to "GENUS" when the taxon was determined to genus level only). | GENUS | 1 | All | Value Genus (=1) only |
| SECTION | Taxon code (was converted to "SECTION" when the taxon was determined to section level). | n/a | 2 | All | Cumulative value Genus (=1)+Section(=1) |
| SPECIES | Taxon code (was converted to "SPECIES" when the taxon was determined to species level). | SPECIES | 3 | All | Cumulative value Genus (=1)+Section (=1)+Species (=1) |
| SUBTAXA | Taxon code (was converted to "SUBSPECIES" when the taxon was determined to subspecies level. | SUBTAXA | 4 | All | Cumulative value Genus (=1)+Section (=1)+Species (=1) and Subspecies (=1) |
| SPAUTHOR | Authority for the species name | SPAUTHOR | 1 | W |  |
| SUBTAUTHOR | Authority for the subspecies name | SUBTAUTHOR | 2 | W | Cumulative value Species (=1) and Subspecies (=1) |
| ACCENUMB | ITC code | ACCENUMB | 1 | All |  |
| ACCENAME | Vernacular name | ACCENAME | 1 | All |  |
| REMARKS | Notes | REMARKS | 1 | All |  |
| DONORNUMB | Code of the accession in the previous location (=donor) | DONORNUMB | 1/Nmax | All | Nmax is the maximum level (1 to 4) of this accession for which a PRVL.LCOD or PRVL.LDET exists. |
| OTHERNUMB1 | Code of the accession in the location before LCOD_N1T1 | OTHERNUMB | 1/Nmax | All | Nmax is the maximum level (1 to 4) of this accession for which a PRVL.LCOD or PRVL.LDET exists. |
| OTHERNUMB2 | Code of the accession in the location before LCOD_N2T1 | n/a | 1/Nmax | All | Nmax is the maximum level (1 to 4) of this accession for which a PRVL.LCOD or PRVL.LDET exists. |
| OTHERNUMB3 | Code of the accession in the location before LCOD_N3T1 | n/a | 1/Nmax | All | Nmax is the maximum level (1 to 4) of this accession for which a PRVL.LCOD or PRVL.LDET exists. |
| DONORDESCR | Donor name description | DONORDESCR | 1/Nmax | All | Nmax is the maximum level (1 to 4) of this accession for which a PRVL.LCOD or PRVL.LDET exists. |
| DONORDESCR2 | Donor description of the accession in the location before LDET_N1T1 | n/a | 1/Nmax | All | Nmax is the maximum level (1 to 4) of this accession for which a PRVL.LCOD or PRVL.LDET exists. |
| DONORDESCR3 | Donor description of the accession in the location before LDET_N2T1 | n/a | 1/Nmax | All | Nmax is the maximum level (1 to 4) of this accession for which a PRVL.LCOD or PRVL.LDET exists. |
| DONORDESCR4 | Donor description of the accession in the location before LDET_N3T1 | n/a | 1/Nmax | All | Nmax is the maximum level (1 to 4) of this accession for which a PRVL.LCOD or PRVL.LDET exists. |
| BREEDMTD | Breeding method used | n/a | 1 | BM+MC |  |
| BREDCODE | Breeding institute code from table Institute | BREDCODE | 1 | BM+MC |  |
| PARFEM | Female parent of hybrid | ANCEST | 1 | BM+MC |  |
| PARMAL | Male parent of hybrid | ANCEST | 1 | BM+MC |  |
| ANNDIF | Registration year of hybrid | n/a | 1 | BM+MC |  |
| BREEDNOT | Remarks on breeding | n/a | 1 | BM+MC |  |
| LOSTELIM | Flag to indicate if Accession 0 = Active 1 = Eliminated 2 = Lost | n/a | 1 | All |  |
| LOSTDATE | Loss date of accession | n/a | 1 | Only for lost/ eliminated accessions |  |
| LOSTNOTE | Note on lost accession/elimination | n/a | 1 | Only for lost/ eliminated accessions |  |
| COLLSITE | Description of collecting site | COLLSITE | 1 | W+TC | The (x) can vary between 1 and 5, but this "T2" type field, which refers to a collecting type of event, can only occur once per accession. |
| COLLDATE | Collecting date | COLLDATE | 1 | W+TC |  |
| ELEVATION | Elevation collecting site | ELEVATION | 1 | W+TC |  |
| COLLNUMB | Collecting number | COLLNUMB | 1 | W+TC |  |
| LATITUDE | Latitude of collecting site (decimal value) | LATITUDE | 1 | W+TC |  |
| LONGITUDE | Longitude of collecting site (decimal value) | LONGITUDE | 1 | W+TC |  |
| CROPNAME | Common crop name | CROPNAME | 0 |  |  |
| DUPLSITE | Location of safety duplicates | DUPLSITE | 0 |  |  |
| MLSSTAT | Multi Lateral System status of the accession | MLSSTAT | 0 |  |  |
| STORAGE | Type of germplasm storage | STORAGE | 1 |  |  |
| ACCEURL |  |  | 0 |  | Link to accession page available on website |

1. All= All population types, W+TC= Wild germplasm and Traditional Cultivars, BM+MC= Breeders Material and Modern Cultivars [↑](#footnote-ref-1)
